# *N*-[4-Chloro-3-(trifluoro­meth­yl)phen­yl]-2,2-dimethyl­propanamide

**DOI:** 10.1107/S1600536812031650

**Published:** 2012-07-25

**Authors:** Yu Zhou, Lili Ren, Yongyu Lu, Feng Zhang, Guoguang Chen

**Affiliations:** a School of Pharmaceutical Sciences, Nanjing University of Technology, Puzhu South Road No. 30 Nanjing, Nanjing 210009, People’s Republic of China

## Abstract

In the title compound, C_12_H_13_ClF_3_NO, the C—C—N—C torsion angle between the benzene ring and the pivaloyl group is −33.9 (5)°. In the crystal, molecules are linked *via* N—H⋯O hydrogen bonds to form chains running parallel to the *c* axis. Weak van der Waals inter­actions are also observed.

## Related literature

For background information on related compounds, see: Rosenblum *et al.* (1998 ▶); Wang *et al.* (2009 ▶). For a related crystal structure, see: Zhu *et al.* (2007 ▶).
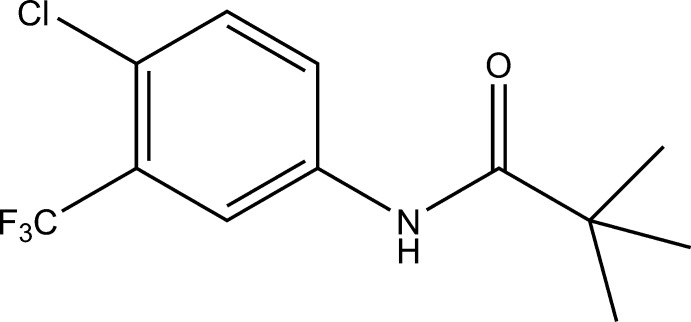


## Experimental

### 

#### Crystal data

C_12_H_13_ClF_3_NO
*M* *_r_* = 279.68
Monoclinic, 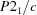

*a* = 5.8850 (12) Å
*b* = 21.955 (4) Å
*c* = 10.307 (2) Å
β = 104.50 (3)°
*V* = 1289.3 (5) Å^3^
*Z* = 4
Mo *K*α radiation
μ = 0.32 mm^−1^
*T* = 293 K
0.30 × 0.20 × 0.10 mm

#### Data collection

Enraf–Nonius CAD-4 diffractometer
Absorption correction: ψ scan (North *et al.*, 1968 ▶) *T*_min_ = 0.910, *T*_max_ = 0.969
2599 measured reflections
2364 independent reflections
1477 reflections with *I* > 2σ(*I*)
*R*_int_ = 0.052
3 standard reflections every 200 reflections intensity decay: 1%

#### Refinement

*R*[*F* ^2^ > 2σ(*F* ^2^)] = 0.061
*wR*(*F* ^2^) = 0.192
*S* = 1.00
2364 reflections
163 parameters
H-atom parameters constrained
Δρ_max_ = 0.38 e Å^−3^
Δρ_min_ = −0.38 e Å^−3^

### 

Data collection: *CAD-4 EXPRESS* (Enraf–Nonius, 1989 ▶); cell refinement: *CAD-4 EXPRESS*; data reduction: *XCAD4* (Harms & Wocadlo, 1995 ▶); program(s) used to solve structure: *SHELXS97* (Sheldrick, 2008 ▶); program(s) used to refine structure: *SHELXL97* (Sheldrick, 2008 ▶); molecular graphics: *SHELXTL* (Sheldrick, 2008 ▶); software used to prepare material for publication: *PLATON* (Spek, 2009 ▶).

## Supplementary Material

Crystal structure: contains datablock(s) global, I. DOI: 10.1107/S1600536812031650/pk2427sup1.cif

Structure factors: contains datablock(s) I. DOI: 10.1107/S1600536812031650/pk2427Isup2.hkl

Supplementary material file. DOI: 10.1107/S1600536812031650/pk2427Isup3.cml

Additional supplementary materials:  crystallographic information; 3D view; checkCIF report

## Figures and Tables

**Table 1:** Hydrogen-bond geometry (Å, °)

*D*—H⋯*A*	*D*—H	H⋯*A*	*D*⋯*A*	*D*—H⋯*A*
N—H0*A*⋯O	0.86	2.24	3.041 (4)	155

**Fig. 1.**
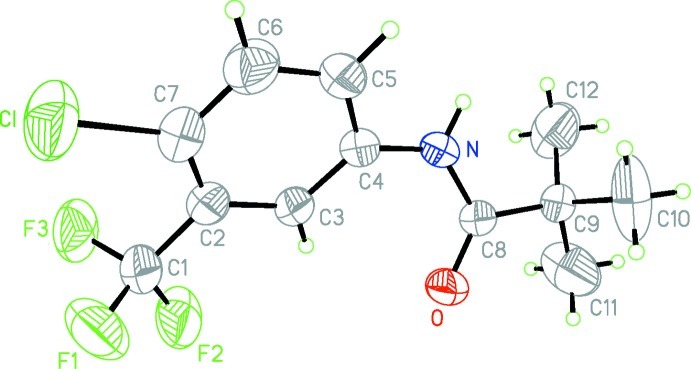
The molecular structure of (I). Displacement ellipsoids are drawn at the 50% probability level. H atoms are shown as small spheres of arbitrary radius.

**Fig. 2.**
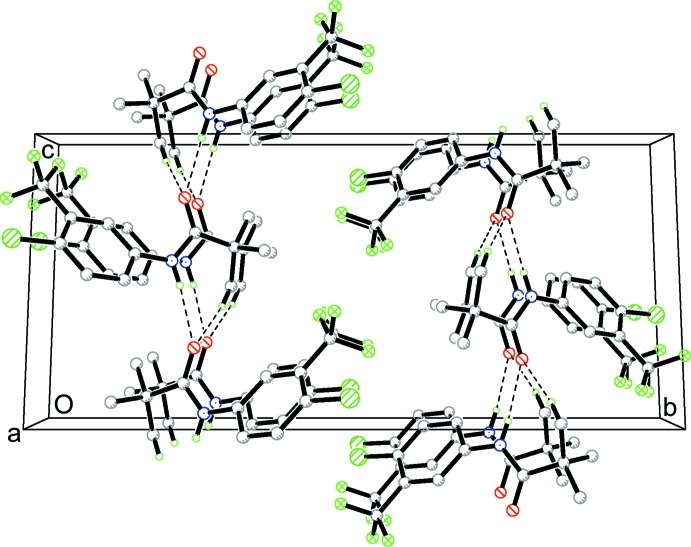
A packing plot of (I), viewed down the a-axis of the unit cell.

## supplementary crystallographic information

### Comment

Ezetimibe is a biologically active molecule, and research has shown it to have the useful property of inhibiting the absorption of cholesterol in the intestine (Rosenblum *et al.*, 1998). As part of our studies into the synthesis of Ezetimibe, the title compound, 4-chloro-3-(trifluoromethyl)-N-pivaloylaniline (I), which is a derivate formed as an intermediate, was synthesized (Wang *et al.*, 2009). In the crystal structure, N—H···O hydrogen bonding interactions (Table 1) link the molecules (Fig. 2) into chains running parallel to the *c* axis.

### Experimental

4-chloro-3-(trifluoromethyl)aniline (C_7_H_5_ClF_3_N, 23.40 g, 0.12 mol) in CH_2_Cl_2_ (40 ml) was added to 4-dimethylaminopyridine (C_7_H_10_N_2_, 1.2 g, 0.01 mol), and Et_3_N (42.3 ml, 0.31 mol) and the reaction was cooled to 273 K. A solution of pivaloyl chloride (C_5_H_9_ClO, 14.4 g, 0.12 mol) in CH_2_Cl_2_ (150 ml) was added dropwise over 1 h and the mixture was then heated to reflux. After 12 h, H_2_O and H_2_SO_4_ (2 N, 75 ml) were added, the layers were separated, and the organic layer was washed sequentially with NaOH (10%), NaCl (satd) and water. The organic layer was dried over MgSO_4_ and concentrated to obtain the product as a pure yellow solid (Wang *et al.*, 2009). Crystals suitable for X-ray diffraction were obtained by slow evaporation of an ethanolic solution.

### Refinement

All H atoms were positioned geometrically and refined using a riding model, with C—H = 0.93 and 0.97 Å, for aryl and methylene H-atoms respectively, and 0.86 Å for N—H. The *U*_iso_(H) were included at 1.5*U*_eq_(C) for the methyl groups and 1.2*U*_eq_ for all other hydrogen atoms.

### Crystal data

**Table:**

C_12_H_13_ClF_3_NO	*F*(000) = 576
*M**_r_* = 279.68	*D*_x_ = 1.441 Mg m^−^^3^
Monoclinic, *P*2_1_/*c*	Mo *K*α radiation, λ = 0.71073 Å
Hall symbol: -P 2ybc	Cell parameters from 25 reflections
*a* = 5.8850 (12) Å	θ = 10–13°
*b* = 21.955 (4) Å	µ = 0.32 mm^−^^1^
*c* = 10.307 (2) Å	*T* = 293 K
β = 104.50 (3)°	Block, colorless
*V* = 1289.3 (5) Å^3^	0.30 × 0.20 × 0.10 mm
*Z* = 4	

### Data collection

**Table:**

Enraf–Nonius CAD-4 diffractometer	1477 reflections with *I* > 2σ(*I*)
Radiation source: fine-focus sealed tube	*R*_int_ = 0.052
Graphite monochromator	θ_max_ = 25.4°, θ_min_ = 1.9°
ω/2θ scans	*h* = 0→7
Absorption correction: ψ scan (North *et al.*, 1968)	*k* = 0→26
*T*_min_ = 0.910, *T*_max_ = 0.969	*l* = −12→12
2599 measured reflections	3 standard reflections every 200 reflections
2364 independent reflections	intensity decay: 1%

### Refinement

**Table:**

Refinement on *F*^2^	Primary atom site location: structure-invariant direct methods
Least-squares matrix: full	Secondary atom site location: difference Fourier map
*R*[*F*^2^ > 2σ(*F*^2^)] = 0.061	Hydrogen site location: inferred from neighbouring sites
*wR*(*F*^2^) = 0.192	H-atom parameters constrained
*S* = 1.00	*w* = 1/[σ^2^(*F*_o_^2^) + (0.1*P*)^2^ + 0.6*P*] where *P* = (*F*_o_^2^ + 2*F*_c_^2^)/3
2364 reflections	(Δ/σ)_max_ < 0.001
163 parameters	Δρ_max_ = 0.38 e Å^−^^3^
0 restraints	Δρ_min_ = −0.38 e Å^−^^3^

### Special details

**Table:**

Geometry. All e.s.d.'s (except the e.s.d. in the dihedral angle between two l.s. planes) are estimated using the full covariance matrix. The cell e.s.d.'s are taken into account individually in the estimation of e.s.d.'s in distances, angles and torsion angles; correlations between e.s.d.'s in cell parameters are only used when they are defined by crystal symmetry. An approximate (isotropic) treatment of cell e.s.d.'s is used for estimating e.s.d.'s involving l.s. planes.
Refinement. Refinement of *F*^2^ against ALL reflections. The weighted *R*-factor *wR* and goodness of fit *S* are based on *F*^2^, conventional *R*-factors *R* are based on *F*, with *F* set to zero for negative *F*^2^. The threshold expression of *F*^2^ > σ(*F*^2^) is used only for calculating *R*-factors(gt) *etc*. and is not relevant to the choice of reflections for refinement. *R*-factors based on *F*^2^ are statistically about twice as large as those based on *F*, and *R*- factors based on ALL data will be even larger.

### Fractional atomic coordinates and isotropic or equivalent isotropic displacement parameters (Å^2^)

**Table:**

	*x*	*y*	*z*	*U*_iso_*/*U*_eq_	
Cl	0.8303 (2)	0.49337 (6)	0.12942 (14)	0.0873 (5)	
O	0.1944 (5)	0.25926 (12)	0.2729 (2)	0.0647 (8)	
N	0.2134 (5)	0.27644 (13)	0.0605 (3)	0.0494 (7)	
H0A	0.1632	0.2645	−0.0211	0.059*	
F1	0.7046 (6)	0.47123 (13)	0.3967 (3)	0.1021 (10)	
C1	0.4957 (8)	0.46500 (17)	0.3095 (4)	0.0639 (11)	
F2	0.3471 (6)	0.44832 (12)	0.3808 (3)	0.1073 (11)	
C2	0.5006 (6)	0.42126 (16)	0.1992 (3)	0.0483 (9)	
F3	0.4298 (5)	0.52091 (10)	0.2663 (3)	0.0797 (8)	
C3	0.3567 (6)	0.37039 (15)	0.1826 (3)	0.0474 (9)	
H3A	0.2576	0.3643	0.2389	0.057*	
C4	0.3598 (6)	0.32856 (15)	0.0826 (3)	0.0444 (8)	
C5	0.5033 (7)	0.33915 (17)	−0.0024 (3)	0.0540 (9)	
H5A	0.5024	0.3120	−0.0718	0.065*	
C6	0.6482 (7)	0.38958 (19)	0.0147 (4)	0.0623 (11)	
H6A	0.7468	0.3957	−0.0420	0.075*	
C7	0.6471 (6)	0.43065 (17)	0.1149 (4)	0.0542 (9)	
C8	0.1439 (6)	0.24321 (15)	0.1556 (3)	0.0442 (8)	
C9	0.0085 (6)	0.18476 (15)	0.1083 (3)	0.0463 (8)	
C10	0.1853 (9)	0.1369 (2)	0.0953 (7)	0.107 (2)	
H10A	0.2984	0.1321	0.1798	0.161*	
H10B	0.2638	0.1493	0.0284	0.161*	
H10C	0.1063	0.0989	0.0694	0.161*	
C11	−0.1150 (11)	0.1652 (3)	0.2130 (5)	0.110 (2)	
H11A	−0.0022	0.1605	0.2976	0.165*	
H11B	−0.1931	0.1270	0.1872	0.165*	
H11C	−0.2283	0.1955	0.2210	0.165*	
C12	−0.1714 (9)	0.1921 (2)	−0.0249 (5)	0.0984 (18)	
H12A	−0.0938	0.2042	−0.0923	0.148*	
H12B	−0.2838	0.2226	−0.0166	0.148*	
H12C	−0.2504	0.1540	−0.0500	0.148*	

### Atomic displacement parameters (Å^2^)

**Table:**

	*U*^11^	*U*^22^	*U*^33^	*U*^12^	*U*^13^	*U*^23^
Cl	0.0823 (8)	0.0705 (8)	0.1155 (10)	−0.0271 (6)	0.0370 (7)	−0.0055 (7)
O	0.107 (2)	0.0532 (16)	0.0354 (13)	−0.0215 (15)	0.0202 (13)	−0.0028 (11)
N	0.074 (2)	0.0427 (16)	0.0316 (13)	−0.0087 (14)	0.0141 (13)	−0.0051 (12)
F1	0.136 (3)	0.078 (2)	0.0703 (16)	0.0004 (17)	−0.0147 (17)	−0.0234 (14)
C1	0.095 (3)	0.043 (2)	0.055 (2)	−0.011 (2)	0.021 (2)	−0.0062 (18)
F2	0.196 (3)	0.0666 (17)	0.0886 (18)	−0.0380 (19)	0.090 (2)	−0.0322 (14)
C2	0.062 (2)	0.0390 (19)	0.0420 (18)	0.0023 (16)	0.0098 (16)	0.0041 (15)
F3	0.105 (2)	0.0383 (13)	0.0994 (18)	0.0013 (12)	0.0319 (15)	−0.0087 (12)
C3	0.068 (2)	0.0380 (19)	0.0395 (17)	−0.0036 (16)	0.0199 (16)	0.0009 (14)
C4	0.060 (2)	0.0382 (18)	0.0355 (17)	0.0003 (15)	0.0126 (15)	0.0012 (14)
C5	0.074 (2)	0.046 (2)	0.0458 (19)	0.0035 (18)	0.0225 (18)	−0.0040 (16)
C6	0.070 (3)	0.060 (2)	0.067 (2)	−0.004 (2)	0.035 (2)	0.002 (2)
C7	0.058 (2)	0.043 (2)	0.061 (2)	−0.0044 (17)	0.0159 (18)	0.0035 (18)
C8	0.060 (2)	0.0380 (18)	0.0351 (18)	0.0023 (15)	0.0136 (15)	0.0013 (14)
C9	0.057 (2)	0.0368 (18)	0.0441 (18)	−0.0022 (15)	0.0118 (15)	0.0005 (15)
C10	0.082 (3)	0.048 (3)	0.186 (6)	0.002 (2)	0.021 (4)	−0.032 (3)
C11	0.144 (5)	0.113 (4)	0.088 (3)	−0.075 (4)	0.056 (3)	−0.027 (3)
C12	0.106 (4)	0.073 (3)	0.090 (3)	−0.028 (3)	−0.026 (3)	0.009 (3)

### Geometric parameters (Å, º)

**Table:**

Cl—C7	1.732 (4)	C6—C7	1.372 (5)
O—C8	1.222 (4)	C6—H6A	0.9300
N—C8	1.364 (4)	C8—C9	1.525 (5)
N—C4	1.416 (4)	C9—C11	1.506 (6)
N—H0A	0.8600	C9—C10	1.508 (6)
F1—C1	1.336 (5)	C9—C12	1.518 (5)
C1—F2	1.326 (5)	C10—H10A	0.9600
C1—F3	1.330 (4)	C10—H10B	0.9600
C1—C2	1.494 (5)	C10—H10C	0.9600
C2—C7	1.385 (5)	C11—H11A	0.9600
C2—C3	1.386 (5)	C11—H11B	0.9600
C3—C4	1.384 (4)	C11—H11C	0.9600
C3—H3A	0.9300	C12—H12A	0.9600
C4—C5	1.379 (5)	C12—H12B	0.9600
C5—C6	1.382 (5)	C12—H12C	0.9600
C5—H5A	0.9300		
			
C8—N—C4	126.6 (3)	O—C8—N	120.9 (3)
C8—N—H0A	116.7	O—C8—C9	122.5 (3)
C4—N—H0A	116.7	N—C8—C9	116.6 (3)
F2—C1—F3	105.3 (4)	C11—C9—C10	109.4 (4)
F2—C1—F1	106.2 (3)	C11—C9—C12	109.0 (4)
F3—C1—F1	105.8 (3)	C10—C9—C12	109.5 (4)
F2—C1—C2	112.6 (3)	C11—C9—C8	108.6 (3)
F3—C1—C2	113.4 (3)	C10—C9—C8	107.3 (3)
F1—C1—C2	112.8 (4)	C12—C9—C8	113.0 (3)
C7—C2—C3	119.9 (3)	C9—C10—H10A	109.5
C7—C2—C1	121.1 (3)	C9—C10—H10B	109.5
C3—C2—C1	119.0 (3)	H10A—C10—H10B	109.5
C4—C3—C2	120.4 (3)	C9—C10—H10C	109.5
C4—C3—H3A	119.8	H10A—C10—H10C	109.5
C2—C3—H3A	119.8	H10B—C10—H10C	109.5
C5—C4—C3	119.0 (3)	C9—C11—H11A	109.5
C5—C4—N	118.6 (3)	C9—C11—H11B	109.5
C3—C4—N	122.3 (3)	H11A—C11—H11B	109.5
C4—C5—C6	120.7 (3)	C9—C11—H11C	109.5
C4—C5—H5A	119.6	H11A—C11—H11C	109.5
C6—C5—H5A	119.6	H11B—C11—H11C	109.5
C7—C6—C5	120.2 (3)	C9—C12—H12A	109.5
C7—C6—H6A	119.9	C9—C12—H12B	109.5
C5—C6—H6A	119.9	H12A—C12—H12B	109.5
C6—C7—C2	119.7 (3)	C9—C12—H12C	109.5
C6—C7—Cl	117.8 (3)	H12A—C12—H12C	109.5
C2—C7—Cl	122.4 (3)	H12B—C12—H12C	109.5
			
F2—C1—C2—C7	−179.3 (4)	C5—C6—C7—C2	0.2 (6)
F3—C1—C2—C7	61.3 (5)	C5—C6—C7—Cl	−179.2 (3)
F1—C1—C2—C7	−59.1 (5)	C3—C2—C7—C6	0.3 (5)
F2—C1—C2—C3	0.2 (5)	C1—C2—C7—C6	179.8 (4)
F3—C1—C2—C3	−119.2 (4)	C3—C2—C7—Cl	179.7 (3)
F1—C1—C2—C3	120.5 (4)	C1—C2—C7—Cl	−0.8 (5)
C7—C2—C3—C4	0.5 (5)	C4—N—C8—O	4.8 (5)
C1—C2—C3—C4	−179.0 (3)	C4—N—C8—C9	−173.3 (3)
C2—C3—C4—C5	−1.8 (5)	O—C8—C9—C11	19.1 (5)
C2—C3—C4—N	−179.0 (3)	N—C8—C9—C11	−162.8 (4)
C8—N—C4—C5	148.6 (3)	O—C8—C9—C10	−99.1 (5)
C8—N—C4—C3	−34.1 (5)	N—C8—C9—C10	79.0 (4)
C3—C4—C5—C6	2.3 (5)	O—C8—C9—C12	140.2 (4)
N—C4—C5—C6	179.6 (3)	N—C8—C9—C12	−41.7 (5)
C4—C5—C6—C7	−1.5 (6)		

### Hydrogen-bond geometry (Å, º)

**Table:**

*D*—H···*A*	*D*—H	H···*A*	*D*···*A*	*D*—H···*A*
N—H0*A*···O	0.86	2.24	3.041 (4)	155
